# Molecular biology of pancreatic neuroendocrine tumors: From mechanism to translation

**DOI:** 10.3389/fonc.2022.967071

**Published:** 2022-09-28

**Authors:** Xiaofei Shen, Xingzhou Wang, Xiaofeng Lu, Yang Zhao, Wenxian Guan

**Affiliations:** ^1^ Department of General Surgery, Affiliated Drum Tower Hospital, Nanjing University Medical School, Nanjing, China; ^2^ State Key Laboratory of Membrane Biology, Institute of Zoology, Chinese Academy of Sciences, Beijing, China

**Keywords:** pancreatic neuroendocrine tumors (pancreatic NETs), molecular biology, epigenetic modulation, targeted therapies, signaling pathway

## Abstract

Pancreatic neuroendocrine tumors (pNETs) are a group of heterogeneous tumors originated from progenitor cells. As these tumors are predominantly non-functional, most of them display asymptomatic characteristics, making it difficult to be realized from early onset. Therefore, patients with pNETs are usually diagnosed with metastatic disease or at a late disease stage. The relatively low incidence also limits our understanding of the biological background of pNETs, which largely impair the development of new effective drugs. The fact that up to 10% of pNETs develop in patients with genetic syndromes have promoted researchers to focus on the gene mutations and driver mutations in *MEN1*, *DAXX*/*ATRX* and mTOR signaling pathway genes have been implicated in disease development and progression. Recent advances in sequencing technologies have further enriched our knowledge of the complex molecular landscape of pNETs, pointing out crucial roles of genes in DNA damage pathways, chromosomal and telomere alterations and epigenetic dysregulation. These novel findings may not only benefit early diagnosis of pNETs, but also help to uncover tumor heterogeneity and shape the future of translational medical treatment. In this review, we focus on the current molecular biology of pNETs and decipher how these findings may translate into future development of targeted therapy.

## Introduction

Neuroendocrine tumors are tumors originating from progenitor cells with neuroendocrine functions, which are a large group of cells that have a neuroendocrine phenotype in the body with the capacity of producing a variety of hormones. Pancreatic neuroendocrine tumors (pNETs) are types of neuroendocrine tumors with significant heterogeneity originating from hormone-producing cells (islet cells), accounting for about 4% to 5% of pancreatic primary tumors (1). Although neuroendocrine tumors can arise from any location of the gastrointestinal tract and bronchopulmonary system, with small intestinal NETs being firstly discovered in the early 20^th^ century, increasing evidence has shown that pNETs are actually a distinct biological disease possessing unique genetic phenotypes (2). pNETs are further classified into functional (hormone producing) and nonfunctional tumors based on the secretion of hormones, and nonfunctional pNETs account for 85% of pNETs, with a significantly worse prognosis compared to functional pNETs (2). Therefore, these asymptomatic pNETs are often detected at advanced stages when patients have missed the best opportunity for treatment, resulting in the overall poor prognosis of pNETs.

In the past 30 years, with the application of imaging, endoscopy, and biomarker detection technologies, the detection rate of pNETs have increased significantly (3), whereas overall survival has remained relatively unchanged during the past several decades (1, 2). Similar to other solid tumors, pNETs also display tumor heterogeneity which leads to the difficulty in targeted therapy development. The tumor heterogeneity is mediated by different molecular biological mechanisms, which is also an important reason for the different tumor behaviors and symptoms of different types of pNETs. According to the different pathological behavior, the World Health Organization (WHO) proposed a common framework to describe the classification of pNETs (**Table 1**) (4). Furthermore, according to the different genetic and epigenetic signatures, the pNETs can be divided into six subtypes, each corresponding to different prognosis and pathological features (5). The genetic characteristics of well-differentiated nonfunctional-pNETs and low-differentiated pancreatic neuroendocrine carcinoma (pNEC) are largely different, which lead to differences in the formation, growth and progression of tumors. Poorly differentiated pNEC often has mutations in the *TP53* and *RB1* genes, resulting in pNEC with strong invasion and metastasis ability, and genetic changes such as *ATRX*/*DAXX* and *MEN1* are more common in patients with highly differentiated nonfunctional-pNETs (6, 7). Since deep insights on alteration in molecular biology stand a chance to provide novel strategies on targeting therapy towards malignancies, our research group has conducted a series of great exploration on malignant lesions (8–10). However, relatively limited understanding of the molecular control of pNETs development also slows down the pace to new effective therapies. With the rapid development of next generation sequencing (NGS), our understanding of the molecular mechanism and genotyping of pNETs has accelerated (11). A large number of studies have found that the occurrence and development of pNETs are associated with epigenetic abnormalities such as gene mutations, DNA damage repair, DNA methylation, histone modification, chromosome remodeling, and activation of alternative lengthening of telomeres (ALT) mechanisms and related signal pathways. This review summarizes current advances in molecular biology of pNETs tumorigenesis and provides future prospects in targeted therapy development based on these molecular alterations.

**Table 1:** The World Health Organization (WHO) Gastrointestinal and pancreatobiliary tract neuroendocrine neoplasms classification.

Neuroendocrine neoplasm type	Classification	Diagnostic Criteria
Neuroendocrine tumor (NET)	NET, grade 1	< 2 mitoses/2 mm^2^ and/or Ki67 < 3%
	NET, grade 2	2-20 mitoses/2 mm^2^ and/or Ki67 3-20%
	NET, grade 3	> 20 mitoses/2 mm^2^ and/or Ki67 > 20%
Neuroendocrine Carcinoma (NEC)	Small cell NEC	> 20 mitoses/2 mm^2^ and/or Ki67 > 20%, with small cell cytomorphology
	Large cell NEC	> 20 mitoses/2 mm^2^ and/or Ki67 > 20%, with large cell cytomorphology

## Molecular control of pNETs in genetic syndromes

A total of 10% pNETs occurs in genetic syndromes. In-depth research on genetic syndromes provides a basis for further understanding of genetic changes during tumor formation (1, 12). Multiple endocrine tumor syndrome type I (MEN1): Hereditary pNETs is the most common in MEN1 syndrome, seen in about 30% to 80% of patients with MEN1. Most of pNETs in MEN1 are small multiple non-functional tumors with a well-differentiated phenotype. MEN1 syndrome is caused by *MEN1* tumor suppressor gene germline inactivation mutation and normal allele somatic cell loss. The mutation can lead to the loss of the expression of its coding protein-menin protein. As a tumor suppressor, menin protein can interact with more than 40 proteins, and participate in gene transcription regulation, chromosome stability, DNA repair, epigenetic regulation and other processes to regulate cell proliferation (13). In pNETs patients with multiple endocrine tumors, the menin protein may be modified by the histone modification of the cyclin B2 promoter region, which affects histone H3 acetylation and H3K4me3 methylation levels and thus plays a regulatory role (14). The *MEN1* gene is also the most common mutant gene in sporadic pNETs, which plays an important role in tumorigenesis (2, 6). VHL disease (Von Hippel-Lindau disease, VHL): The incidence of pNETs in VHL disease is around 15%. Generally, it is a small non-functioning tumor with multiple occurrences. Liver metastasis is rare, and the prognosis is better than sporadic cases. *VHL* gene inactivation mutations can inhibit the ubiquitination of hypoxia-inducible factor transcription factors, leading to increased expression of target genes related to hypoxia-driven angiogenesis pathways, and promoting angiogenesis and tumor growth. *VHL* gene mutation is extremely rare in sporadic pNETs, but it can cause similar gene mutation effects through gene censorship or gene promoter hypermethylation (3, 15). Neurofibromatosis type I (NF1): The incidence of pNETs in NF1 patients is less than 10%. *NF1* gene encodes neurofibrillin, which is a negative regulator of RAS/MAPK and PI3K/AKT/mTOR signal transduction network. Inactivation mutation of *NF1* gene causes an increase in the activity of related pathways, which may promote tumor progression to malignancy (15). (4) Tuberous sclerosis (TSC): pNETs is extremely rare in TSC, only occurs in 1% of patients, and is mainly a non-functional tumor. The proteins encoded by the *TSC1* and *TSC2* genes can form a complex and function together. *TSC1* or *TSC2* gene inactivation mutations make the TSC complex lose its inhibitory effect on the mTOR pathway and promote cell proliferation (15). In sporadic pNETs, down-regulation and mutation of *TSC1*/*TSC2* genes were also found (6).

## Molecular alteration in sporadic pNETs

Most pNETs are sporadic with a long onset. During the onset of pNETs, molecular alteration including typical gene mutations occurs. Chromosomal changes and gene fusion has also been suggested to play an important role in tumorigenesis. Owing to the development of sequencing technologies, the discovery of various genetic events with molecular heterogeneity provides new spectrum of disease development and progression (11). Related genetic changes in pNETs formation involve several critical signaling pathways and chromatin remodeling, with telomere maintenance and DNA damage also being identified.

### MEN1 mutations

The *MEN1* gene is located at 11q13, and it is highly conserved during evolution. The coding protein of *MEN1* gene is called menin. Menin participates in the regulation of transcription and maintains the homeostasis of the gene group. 90% of patients with germline mutations in the *MEN1* gene will eventually develop multiple endocrine tumor syndrome type I as discussed above. In addition, *MEN1* mutations have been discovered in both functional and non-functional pNETs (6, 7). Loss of menin expression or abnormal nuclear translocation caused by *MEN1* gene mutations will cause a series of signaling pathway disorders, and then result in systemic endocrine diseases including pNETs. Menin interacts with many transcription factors in the cell nucleus, directly or indirectly participates in epigenetic regulation processes such as histone methylation modification and chromosome remodeling, and plays a key regulatory role in the normal transcription of target genes and the maintenance of cell phenotype. Tumor chromosome translocation causes the loss of important domains of chromatin binding protein, leading to the loss of its ability to recruit SWI/SNF complexes, and thus destroying the transcriptional function of chromatin binding protein regulating genes (7). Menin can also recruit protein arginine methyltransferase 5 (PRMT5) to the promoter region of *Gas1* gene, a key factor of the Hedgehog signaling pathway, and strengthen the inhibition of histone arginine methylation, thereby inhibiting the Hedgehog signaling pathway to achieve the anti-tumor effect (16). Interestingly, in different tissues, menin can regulate the transcription of different target genes through the same histone modification mechanism to exert different biological function (17). In addition, menin and DAXX use histone (H3K9me3) modification mechanism to regulate the endopeptidase promoter of membrane metal and affect the occurrence of pNETs (18). The *ATRX*/*DAXX* and *MEN1* genes can maintain the integrity and stability of the somatic cell genome by regulating the structure of chromatin. If there is a problem with these regulatory genes, it will promote the occurrence and development of pNETs. Studies have sequenced 38 well-differentiated pNETs specimens, and also found that *MEN1* gene, *ATRX*/*DAXX* gene and PI3K/AKT/mTOR signaling pathway are the most common hotspot mutations (19).

### ATRX/DAXX mutations in chromatin remodeling and telomere maintenance

Chromatin remodeling related gene mutations are very common in pNETs. The menin protein can recruit the MLL1 histone methyltransferase complex to play an important role in chromatin remodeling and gene expression (13). The discovery of the role of *ATRX* and *DAXX* gene mutations in pNETs is of great significance. DAXX is a specific histone chaperone that can guide histone H3.3 to deposit on the inter-arm and telomere heterochromatin, and ATRX is the chromatin remodeling adenosine triphosphate enzyme in the SWI/SNF family (20). Related proteins encoded by *ATRX*/*DAXX* genes can form complexes and combine with histone H3.3 to deposit them in specific areas such as telomeres and centromeres, and participate in apoptosis and chromatin remodeling (7). In addition, the ATRX-DAXX complex may form nucleosomes containing H3.3 through replication-independent methods, such as defective nucleosomes. It can lead to DNA damage and genome instability. At the same time, at the end of the chromosome, the ATRX/DAXX complex also needs to inhibit the defective DNA for repair, and the complex formed by the mutation cannot inhibit the repair of the defective DNA, so that telomere fusion occurs. *ATRX* gene mutations can also cause chromatin plate assembly, chromatin condensation, and centromeric dysfunction during chromosome mitosis. These abnormal changes can cause chromosomal mutations and lead to the occurrence of pNETs (21).

Tumors maintain the length of telomeres in cells without relying on telomerase, and the mechanism by which cells continue to proliferate is called alternative lengthening of telomeres (ALT) (22). *ATRX*/*DAXX* gene mutations are related to the ALT phenotype, and those with gene mutations have a better prognosis. In tumors> 2 cm, *ATRX*/*DAXX* gene mutations are common, but no related protein expression loss was found in microadenomas, indicating that the loss of ATRX/DAXX protein expression and the appearance of ALT phenotype may occur at the progression phase of pNETs (23). Consistent with these, immunohistochemistry staining on 192 metastases of 52 patients with pNETs showed that 52% metastases had *ATRX*/*DAXX* gene expression loss, with both ATRX/DAXX and ALT abnormalities and a shortened survival (24). Another study found that the frequencies of ATRX/DAXX inactivation and ALT activation in pNETs patients were 19.3% and 20.8%, respectively, which were related to a high-grade tumor, neurovascular invasion, liver metastasis and other malignant clinical pathological features, as well as a prognostic survival (25). Moreover, recent study has shown that loss of *ATRX*/*DAXX* and presence of ALT are able to predict distant metastasis and are correlated to inferior overall survival and relapse-free survival in ≤2.0cm non-functional pNETs (26). Hence, *ATRX/DAXX* status and ALT detection are recommended as prognostic biomarkers for pNETs, especially for non-functional pNETs. *ATRX*/*DAXX* somatic mutations are common in extended telomerase. The shortened telomerase has fewer *ATRX*/*DAXX* mutations, but more chromosome fragmentation and gene fusion. Research has also shown that non-functional pNETs also has copy number abnormalities, accompanied by *DAXX*/*ATRX* gene mutations, and both can accurately predict the risk of postoperative recurrence (27). Most of the frameshift mutations and nonsense mutations of *ATRX* and *DAXX* in pNETs can cause complete loss of protein expression. More importantly, *ATRX*/*DAXX* gene mutations are related to the alpha cell origin of non-functional pNETs, and the prognosis of such patients is worse than that of wild-type gene mutations (28).

### PI3K/AKT/mTOR signaling pathway

**Figure 1:**
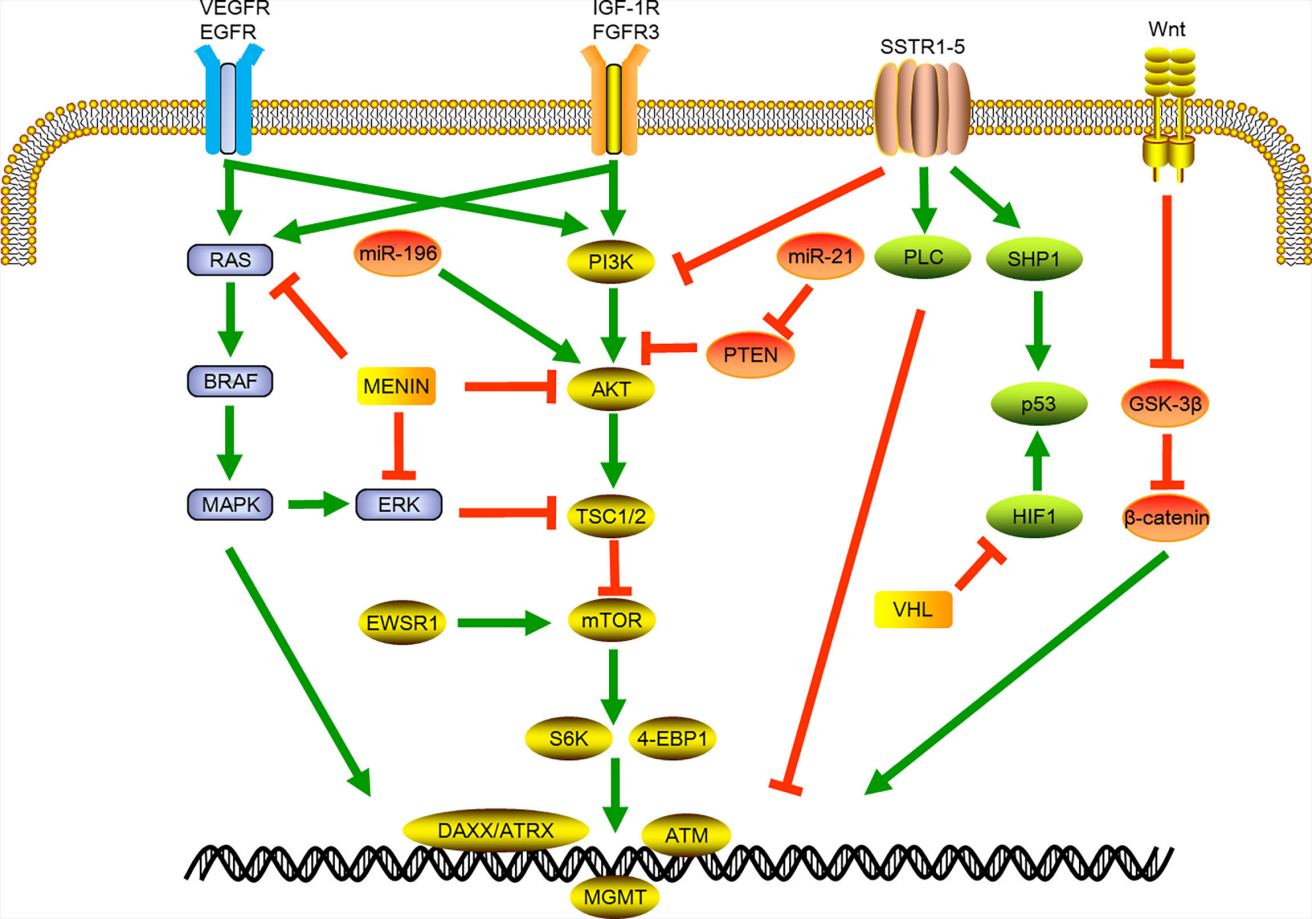
Schematic of signaling pathways involved in pancreatic neuroendocrine tumor development and progression. VEGFR, vascular endothelial growth factor receptor; EGFR, epidermal growth factor receptor; BRAF, B-Raf Proto-oncogene; MAPK, mitogen-activated protein kinase; miR, micro RNA; ERK, Extracellular-signal-regulated kinase; EWSR1, EWS RNA binding protein 1; IGF-1R, insulin-like growth factor receptor 1; FGFR3, fibroblast growth factor receptor 3; PI3K, phosphatidylinositol-3-kinase; AKT, protein kinase B; TSC1/2, Tuberous sclerosis 1; mTOR, mammalian target of rapamycin; S6K,; 4-EBP1, Eukaryotic translation initiation factor 4E-binding protein 1; DAXX, death domain-associated protein; ATRX, X-linked mental retardation and a-thalassemia syndrome protein; ATM, ataxia telangiectasia; SSTR, somatostatin receptor; PTEN, phosphatase and tensin homolog deleted; PLC, phospholipase C; SHP1, Src homology region 2 domain-containing phosphatase-1; HIF1, hypoxia-inducible factor 1; VHL, Von Hippel-Lindau.

Phosphatidylinositol 3-kinase (PI3K) belongs to the lipid kinase family, which can be activated by extracellular growth factor signals to phosphorylate and activate protein kinase B (AKT) and participate in protein synthesis, cell growth, and many other cellular processes. Mammalian target of rapamycin (mTOR) is the most important downstream factor in the PI3K/AKT/mTOR pathway. It can combine with a variety of proteins to form mTOR1/2 complexes through the integration of growth factors and nutritional molecules and other intracellular and extracellular signals (29). In sporadic tumors, multiple mTOR-pathway related genes including *TSC2*, *PTEN* and *PIK3CA* genes were widely altered. The menin protein also binds to AKT, which can further inhibit intracellular positional transfer of AKT and lead to the inhibition of mTOR signaling pathway. The PTEN gene encodes a product that inactivates PI3K through dephosphorylation, and the mutation of this gene can activate mTOR signaling. Recent study showed that about 37% of the samples of sporadic pNETs had *MEN1* gene mutations, and about 13% of the samples had mutually exclusive gene mutations related to the mTOR pathway, including *PTEN*, *TSC1*/*TSC2*. In addition to gene mutations, chromosomal changes and gene fusions related to this pathway were also identified, including the repeated amplification of chromosomal loci of the PI3K activator *PSPN* gene and mTOR regulator *ULK1* gene, and the fusion of *EWSR1* gene with *BEND2* and *FLI1* genes, which can activate mTOR signal and promote tumor formation (6). (**Figure 1**)

The frequency of mutations in genes related to the mTOR signaling pathway in pNETs patients is extremely high (6), and there is usually activation of the mTOR pathway, that is, p-mTOR overexpression, accompanied by loss or down-regulation of PTEN expression, both of which are usually in close relation to biological behavior and poor prognosis (30). These evidences all indicate that mTOR-related signaling pathways might be one of the key targets of pNETs therapy. Everolimus is an mTOR inhibitor. In the RADIANT-2 study, compared with placebo and long-acting octreotide (LAR), taking everolimus and LAR can prolong the median disease free survival of patients with pNETs (31). The RADIANT-3 study showed that the everolimus group prolonged the median disease-free survival of advanced pNETs by 6.4 months compared with the placebo group, which is of great clinical significance (32). Somatostatin analogues (SSA) have been used for the treatment of pNETs. Studies showed that the somatostatin signal functions on the insulin-like growth factor 1/PI3K/mTOR pathway, thereby inhibiting the secretion and proliferation activity of pNETs (33). Studies also suggested that the synergistic mechanism of everolimus and LAR was related to the inhibition of mTOR by everolimus and the down-regulation of insulin-like growth factor 1 by octreotide (33). Taken together, both everolimus and octreotide can inhibit tumor growth through the mTOR signaling pathway.

### DNA damage and repair

*ATM* suppressor genes are involved in a variety of cellular processes related to DNA damage. Bersani et al. found ATM gene mutations in 5.5% of the samples (34), indicating that there were changes related to DNA damage and repair during the occurrence of pNETs. Germline mutations in DNA damage repair related genes *MUTYH*, *CHEK2* and *BRCA2* were also discovered (6), indicating that DNA damage repair related genes may be the initiation event of some genetic syndromes. A study of exome sequencing of sporadic insulinomas found that 30% of the samples had functional mutations in the YY1 (YinYang 1, YY1) transcription factor gene *T372R*, which could increase the transcription activity of YY1 and induce the target genes expression such as mitochondrial genes *IDH3A* and *UCP2* (35). Studies have found that other genes related to changes in DNA expression in insulinomas including *H3F3A*, *KDM6A* and *ATR* genes (36). Therefore, these results suggested targeting DNA damage and repair genes may be a new potential therapy for the treatment of pNETs.

## Sporadic pNECs

According to the histological differentiation characteristics of tumors, G3 tumors were further divided into well differentiated pNETs and poorly differentiated pNECs, which usually have *TP53* or *RB1* gene mutations. Among pNETs, pNECs accounts for about 7.5% of the total, and the 5-year survival rate of patients is less than 7%. The survival time of patients with metastasis was even worse, with a usually less than 1 year survival time (37). Yachida et al. analyzed 19 pNECs samples and found that 57% and 71% of samples had mutations in *TP53* and *RB1* genes, respectively (38). A recent large-sample study of 123 pNECs also found the above changes, but this change was very rare in well-differentiated pNETs (39), which further illustrates that poorly differentiated pNECs were not developed from the progression of well-differentiated tumors to poorly differentiated states. Different genetic changes may occur in the development and progression of pNECs, which needs further studies to illustrate.

## Epigenetic control of pNETs

The occurrence of tumors is a process in which genetic and epigenetic changes are intertwined, which together promote the occurrence and progression of tumors. In pNETs, less than half of the tumors caused by genetic changes such as gene mutations indicating that epigenetic changes have a non-negligible role in tumorigenesis (6). Epigenetic control of pNETs includes CpG methylation, histone modification, and non-coding RNA molecules, which have all been studied extensively in the development and progression of pNETs.

### DNA methylation

DNA methylation mostly occurs in the nucleotides of gene CpG islands and is mediated by DNA methylation transferase. In cancer cells, downregulated gene expression due to promoter hypermethylation is the hallmark of dysregulated CpG methylation which leads to silencing of tumor suppression genes and tumor development. Genome-wide hypomethylation which result in DNA instability is also a typical characteristic during tumor progression. There are many gene methylation changes in pNETs, among which the *RASSF1* (Ras association domain family 1 isoform A) gene, regulated by p53 and DAXX, is the most common hypermethylated gene in metastatic tumors (40). Hypermethylation of *VHL*, *HOPX* and *TIMP3* gene promoter is also related to metastatic tumors with poor prognosis (12, 41, 42). *CDKN2A* gene promoter methylation is associated with early tumor recurrence and shortened overall survival (43). *MGMT* promoter hypermethylation has been found in 40-44% of pNETs with more aggressive tumors and worse prognosis (42). Furthermore, the methylation status of the *MGMT* gene promoter can also be used to predict the therapeutic response of temozolomide (44). Not only hypermethylation is involved in pNETs development and progression, but also global hypomethylation has been shown to contribute to different clinical outcome in pNETs. Choi et al. reported for the first time that there was hypomethylation of *LINE1* and *ALU* gene sequences in pNETs (45). Consistent with these results, further studies showed that hypomethylation was related to poor prognosis and progression of tumors (46).

### Histone modification

Histone is an important part of chromatin, which can package DNA to form nucleosomes. After gene translation, histone modification can regulate the binding ability of genes and transcription factors and maintain chromatin structure. Menin protein regulates histone methylation status by recruiting the MLL1 histone methyltransferase complex, and participates in various cellular processes and chromatin remodeling (47). Menin also mediates H3K9 methylation through recruiting SUV39H1 (Suppressor of variegation 3-9 homolog protein family), which is reduced through introduction of patient-derived *MEN1* mutations into the SUV39H1 interaction domain (48). Loss of menin also leads to H3K4me3 loss, which results in MEN1-like sporadic pancreatic tumors (49). Therefore, menin also controls the development of pNETs through regulating methylation process. The protein encoded by *SETD2* gene is involved in the regulation of histone methylation status and chromatin activity. Disruption of SETD2 function was identified in 81% of primary pNETs with distant metastases (24). *ATRX*/*DAXX* gene encoding protein can bind to histones, change the histone deposition area, and participate in chromatin remodeling (12). Loss of *ATRX* and *DAXX* was also associated with shorter survival in patients with pNETs (24). Furthermore, recent studies also found that phosphorylated histone H3 can be used as an ideal prognostic factor to better predict the survival of pNETs patients (50).

### MicroRNAs

Micro-RNA (miRNAs) are small non-coding RNAs that regulate targeted mRNA, affect the expression of downstream effector proteins, and also regulate post-transcriptional gene expression. Along with long non-coding RNAs (lncRNAs), miRNAs are post-transcriptional gene regulators implicated in tumor development and progression (51). miRNAs functions through their imperfect 5’-base pairing with target mRNA sequences to induce gene silencing, and they also have the capacity of upregulating target genes under specific circumstances, indicating a duel properties in both oncogenic and tumor suppressing (52). miRNAs are relatively stable in circulation and their expression correlates with various clinical characteristics not only in different tumor types but also in subtypes of the same tumor. Therefore, since most pNETs are non-functional and asymptotic, the detection of miRNAs in the peripheral may be promising candidates as diagnosis/prognosis tools.

miR21, which targets and downregulates *PTEN*, has been suggested to be in close relation to higher proliferation and metastatic disease. It also targets *PDCD4*, which is a tumor suppressor, to induce the downregulation of *PDCD4* mRNA in metastatic tumors (53). In addition, miR21 was overexpressed in the circulation of patients with pNETs when compared to that of chronic pancreatitis (54), although information in respect to detailed type and grade of pNETs lacked. Similarly, miR210 can be induced by hypoxia-induced stress (55), which may further facilitate the metastasis of pNETs (56). The expression of miR-103 and miR-107 associated with lack of expression of miR-155 discriminated tumors from normal, and miR204 and miR211 further distinguished functional and non-functional pNETs, as they were restricted to insulinomas (57). Regarding to the distinguish between pancreatic ductal adenocarcinoma and pNETs, miR1290 was identified to differentiate pancreatic ductal adenocarcinoma from pNETs with an AUC of 0.80 (56). Other miRNAs involved in pNETs compared to pancreatic ductal adenocarcinoma were miR584, miR1285, miR550a-5P, and miR1825 (58). As mentioned above, the function of miRNAs largely depends on their molecular control of target genes. Therefore, miRNAs involved in the regulation of pNETs related genes might display different functional properties and may guide future studies based on their target gene characteristics. Consistent with these, three clusters of miRNAs were identified recently, with each cluster displaying distinct gene mutational rates in *mTOR*, *MEN1*/*DAXX*/*ATRX* and DNA damage pathways (59). miR-Cluster-1 tumors were enriched in the MEN1 mRNA subtype, and were composed of non-functional pNETs with a moderate metastatic potential. miR-Cluster-2 tumors were enriched in the metastasis-like primaries mRNA subtype with high metastatic potential, and miR-Cluster-3 tumors were mainly composed of insulinomas.

### Long non-coding RNAs

Studies on the molecular control of pNETs by long non-coding RNAs (lncRNAs) are limited. The HOX antisense intergenic RNA (HOTAIR) and the metastasis-associated lung adenocarcinoma transcript 1 (MALAT1) were newly identified lncRNAs in the control of pNETs development and progression. HOTAIR is a chromatin-modifying lncRNA involved in prostate cancer neuroendocrine differentiation (60), and overexpression of HOTAIR led to increased metastasis of breast cancer cells through manipulating H3K27me (61). Therefore, targeting HOTAIR has been shown to reduce cell invasiveness through the induction of cell cycle arrest and apoptosis (62). However, controversial results were discovered recently in which upregulation of HOTAIR in pNETs resulted in less aggressive disease and better prognosis (63). Similar results were also discovered for MALAT1, which was associated with favorable clinical outcome in pNETs. Detailed functional studies are urged for understanding the related mechanisms. Other dysregulated lncRNAs include MEG3 and lncNEN885 (64, 65). MEG3 was shown to be regulated by menin in cell line model and epigenetic activation of MEG3 was also thought to be an attractive therapeutic approach for potential future clinical use (64). MEG3 directly targets the proto-oncogene c-MET (hepatocyte growth factor receptor) to block cell proliferation and delay cell cycle progression, and it also displays an anti-tumor effect through manipulating the p38/ERK/Akt and Wnt/β-catenin pathway in a MEG3/miR183/BRI3 dependent manner (66).

## Therapy implication

For patients with advanced stage and distant metastasis, comprehensive treatment is the main approach to prolong survival. With a better understanding in molecular biology of pNETs, developing new therapeutic targets based on molecular alterations has been a new promising field. According to the characteristics of the patient’s molecular changes, individualized treatment plans may also better extend the patient’s survival time.

### Somatostatin analogues

Therapy against somatostatin receptor (SSR) is currently most widely used target therapy in pNETs, as around 70% of tumors express somatostatin receptor (67). SSR2 has a high affinity with somatostatin analogues (octreotide or lanreotide, etc.), which can lead to the inhibition of tumor secretion, cell proliferation and angiogenesis. Somatostatin analogs can also be coupled with radionuclide nuclide yttrium or lutetium to treat patients in advanced stage or metastasis. According to reports from clinical trials, the objective response rate of this treatment can reach 20%~60%, with an overall survival period of 53 months (68).

### mTOR inhibitors

As mentioned above, the mTOR pathway is very important in the occurrence of pNETs. Everolimus, an mTOR inhibitor, has been used in patients with advanced tumors (69). However, there is still no reliable diagnostic method for early screening of patients who can benefit from mTOR inhibitor treatment. Since the expression of genes after mutations can be affected by epigenetic changes, screening for mTOR pathway-related proteins or mRNA may help select appropriate patients.

### Anti-angiogenic drugs

The pathological feature of pNETs is characterized by a rich blood supply, and targeted therapy of angiogenic molecules thus may help to inhibit tumor growth. Tyrosine kinase receptor inhibitors have been used for patients with pNETs (70). However, anti-angiogenic drugs may promote tumor progression and distant metastasis, which may be related to tumor hypoxia and the expression of vascular endothelial growth factor A, fibroblast growth factor, ephrin A1, and the activation of the proto-oncogene c-Met (71). Therefore, the combined use of c-Met inhibitors can simultaneously inhibit tumor growth and metastasis. At present, this treatment program has achieved certain effects in animal models, but further clinical studies are still needed. Nitric oxide synthase inhibitors and thrombospondin analogs have also been found in mouse models to inhibit angiogenesis and tumor growth, with potential benefits in clinic in the future (72).

### *MEN1* gene replacement therapy

*MEN1* gene is the most common mutant gene in pNETs, and *MEN1* gene replacement therapy for pNETs with a lack of menin protein expression is gradually emerging. As a tumor suppressive protein, menin, encoded by MEN1, has long been proved to play an anti-tumor role in various tumor when over-expressed in tumor (73, 74). At present, the overexpression of menin protein has been found to inhibit tumor growth in *in vitro* cell line experiments and has been confirmed in animal models of pituitary tumors (75). *MEN1* gene transgene therapy is an emerging and effective treatment method for pNETs with a lack of menin protein expression, but it still needs further confirmation and clinical research.

### Epigenetic regulatory factor

The reversibility of tumor epigenetic changes has a great research value in treatment. Epigenetic regulatory factor is an emerging anti-cancer drug that has been approved for use in diseases such as leukemia (76). In pNETs, patients with methylation of the *MGMT* gene promoter have better therapeutic effects with temozolomide, but this result still needs to be confirmed (77). Studies on histones have found that histone deacetylase (HDACs) inhibitors can enhance the expression of somatostatin receptor mRNA and the uptake of radionuclide-labeled octreotide in pNETs cell lines (QGP1 and BON1), enhancing the effectiveness of related treatment (78). A recent study found that JQ1 (a histone inhibitor) can inhibit tumor growth in the pNETs mouse model (79). At present, most of the apparent regulatory factor drugs for pNETs are in the stage of *in vitro* experiments or animal models, and a large number of clinical studies are still needed to evaluate clinical efficacy.

### Hypoxia-inducible factor

VHL gene mutation can result in pNETs occurrence. It has been found that pNETs caused by VHL disease possibly progress through hypoxia-inducible factor 2a (HIF-2α) and hypoxia related biological process (80). Similarly, a study also found that hypoxia-inducible factor expression was elevated in the pNETs tissue compared to non-tumor samples (81). According to the findings, Belzutifan, a specific HIF-2α inhibitor, has been developed to treat VHL disease and achieved great success, which makes it approved by Food and Drug Administration (FDA) for VHL disease and its related tumor treatment (82).

### Novel molecular for targeting therapy

By using unbiased phage display screening technique, construction of tumor-specific peptides or antibody fragments targeting tumor-associated antigens have become true, thus providing a novel potential therapeutic strategy for malignancies (83). Researchers have found that CDH17, which can be detected in the intestinal epithelial cells, is also highly expressed in pNETs (84). Unbiased phage display screening technique further screened and identified a single variable-domain antibody (VHH) targeting CDH17 in non-functional pNETs, with superiority of better permeability in tissues and reduced toxicity triggered by complement system (85). Based on this finding, the researchers developed chimeric antigen receptor (CAR) T cells targeting CDH17, which achieved eradication in pNETs and showed reliable security in the drug administration (85).

In a word, although pNETs exhibit highly variable clinical characteristics and prognosis, with the increasing awareness of the molecular characteristics of pNETs, more and more precision and translational treatment strategies are being developed. For instance, research into somatostatin receptor and VHL gene mutation have led to application of somatostatin analogues and belzutifan in clinical treatment. The translation of basic research findings into clinical treatment strategies is expected to improve the prognosis of patients with pNETs.

## Conclusion

With the development of genomic sequencing technology, molecular characteristics and tumor heterogeneity of pNETs have been gradually identified, which also help to further classify pNETs and better understand the potential clinical outcomes for patients with different pNETs subtypes. The genetic characteristics of well-differentiated non-functional pNETs and poorly-differentiated pNEC are different. Functional pNETs such as insulinomas also differ in genetic background when compare to non-functional pNETs. In addition, epigenetic changes also play an important role in tumor development and progression, which have an impact on tumor diagnosis, treatment and prognosis. How to integrate various molecular information to fully address disease heterogeneity may not only benefit the diagnosis and classification of pNETs, but also provide promising strategy to develop powerful personalized treatment.

## Author contributions

XS, XW and XL drafted the manuscript. YZ and WG raised the concept and suggested the topic, and YZ illustrated the figures. YZ, WG and XS made contributions in funding acquisition. All authors contributed to the article and approved the submitted version.

## Funding

This work was financially supported by grants from National Natural Science Foundation of China (82172645, 81970500, 81802846), and Natural Science Foundation of Jiangsu Province (BK20180116).

## Conflict of interest

The authors declare that this review is composed in the absence of any commercial or financial relationships that can be constructed as a potential conflict of interest.

## Publisher’s note

All claims expressed in this article are solely those of the authors and do not necessarily represent those of their affiliated organizations, or those of the publisher, the editors and the reviewers. Any product that may be evaluated in this article, or claim that may be made by its manufacturer, is not guaranteed or endorsed by the publisher.
